# Milk production, principal composition and economic assessment of Holstein cows in a silvopastoral system with Mexican sunflower and yacon silage as partial concentrate replacement

**DOI:** 10.1007/s11250-026-05041-6

**Published:** 2026-05-08

**Authors:** Jhon Jairo Lopera-Marín, Joaquín Angulo-Arizala, Enrique Murgueitio Restrepo, Liliana Mahecha-Ledesma

**Affiliations:** 1 Centre for Research on Sustainable Agriculture – CIPAV., Cali, 20591 Colombia; 2 Agricultural Science Research Group - GRICA, University of Antioquia, Medellín, 1226 Colombia

**Keywords:** Cow’s nutrition, Fodder shrubs, Livestock agroforestry systems, Sustainable system

## Abstract

Dairy livestock systems in the Colombian highlands are characterized by grass monoculture, high fertilization, concentrate supplementation, and predominantly Holstein cows. Limited pasture diversity and inadequate management have negatively affected soil and forage quality, increasing dependence on fertilizers and concentrates, raising production cost and reducing profitability. Intensive silvopastoral systems (ISS) represent an agroecological alternative by integrating grasses with trees and shrubs and strategic supplementation to improve production efficiency. Yacon (*S. sonchifolius*), a native Andean Asteraceae, produces high yields of leaves, steams and tubers, making this plant a valuable local resource for supplementation in ISS. This study evaluated milk production, principal milk composition, somatic cell count, and production cost of Holstein cows in an ISS with Mexican sunflower (*T. diversifolia*) and yacon silage as partial replacement for concentrated, compared to Kikuyu grass monoculture supplemented with concentrate. A Latin square crossover design (AB/BA) was used with two evaluation periods, two treatments, and two groups of four cows. Treatments were SPI (ISS + yacon) and MON (kikuyu monoculture). Response variables included total dry matter intake, milk yield, principal milk composition, somatic cells, feed efficiency, energy efficiency, and a descriptive economic analysis. Milk yield or principal composition did not differ between treatments, except for somatic cells (*p < 0.05*), which were lower in SPI. An economic simulation for 50 cows showed annual feed cost savings of USD 4,937.13 with SPI. Results indicate that ISS supplemented with yacon silage can reduce production costs while maintaining milk performance and reducing somatic cell count in dairy systems of the Colombian highlands.

## Introduction

Dairy livestock systems in the tropical highlands of Colombia occupy areas of high plateaus and slopes of the Andes mountain range, ranging from 2,000 to 3,200 m.a.s.l. (Carulla and Ortega 2016; UPRA 2020), following the transformation of original forests, wetlands, and moorland ecosystems (Etter and van Wyngaarden 2000). These systems are characterized by grazing monocultures of Kikuyu grass (*Cenchrus clandestinus* [Hochst. ex Chiov.] Morrone) and hybrid ryegrass (*Lolium hybridum* Hausskn.), with a low presence of creeping legumes, and in some cases without pasture rotation (Benavides Cruz et al. 2021; Lerma-Lasso et al. 2023). Reduced botanical diversity and inadequate pasture management have contributed to adverse effects on the soil properties and forage availability and quality increasing the dependence on synthetic fertilization and concentrate supplementation to sustain the milk production (Durana et al. 2023; Reyes-Palomino and Cano-Ccoa 2022; Lerma-Lasso et al. 2023). This dependence has resulted in higher production cost, compromising the profitability and long-term viability of these dairy production systems (Portillo-López et al. 2019; Arias Segura et al. 2025). In this context, intensive silvopastoral systems (ISS) has been proposed as an agroecological alternative to improve production efficiency through greater functional diversity (Lopera et al. 2015), combining grasses with tree and shrub fodder species (Murgueitio et al. 2011) and strategic supplementation with local forage resources (Meza and Rodríguez 2021).

Among locally available forage resources, Mexican sunflower (*Tithonia diversifolia*) has demonstrated high biomass production, protein, soluble carbohydrates, minerals, and an adequate amount of secondary metabolites, which may improve nutritional efficiency under tropical highland conditions, making it suitable for inclusion in ISS (Lopera-Marín et al. 2025a). In addition, yacon (*Smallanthus sonchifolius* [Poepp.] H. Rob.), a native Andean Asteraceae, not only produces high leaf, steam, and tuber yields (147 t/ha/year fresh basis; Lopera-Marín et al. 2020), but also offers a nutritionally rich forage with high digestibility and sugar content (Lopera-Marín et al. 2025b). While the nutritional quality of yacon and the benefits of ISS have been studied separately, its use as silage to supplement dairy cows in intensive silvopastoral systems has not been previously reported, which motivated this study.

The objective of this study was to evaluate milk yield, principal milk composition, somatic cell count, and production cost of Holstein cows management under an ISS with *T. diversifolia* and yacon silage (*S. sonchifolius*) as a partial replacement for balanced feed compared to fertilized kikuyu grass monoculture in the tropical highlands of Colombia.

## Materials and methods

### Location

The production systems were evaluated in Colombia at the La Montaña Center for Agricultural Practices and Development at the University of Antioquia. It is located in the municipality of San Pedro de los Milagros (Antioquia), at coordinates 6° 26’ 59.60” N and 75° 32’ 37.08’ W. The area belongs to the low montane humid forest zone (Holdridge 1966), with annual precipitation of 1575 mm, an average temperature of 15°C, relative humidity of 72%, and an altitude of 2350 meters above sea level.

### Experimental design and assignment of evaluation sequences

All data were collected using a Latin square design with a crossover (AB/BA sequence), with two evaluations, two treatments per evaluation, and two groups of cows (G1 and G2) for each treatment and evaluation. Eight Holstein-Friesian cows were selected and assigned to two evaluations (four cows per evaluation) carried out at different times. In each evaluation, two groups were defined with four experimental units per group. The experiment lasted 70 days, during which 35 days per evaluation and two experimental periods per evaluation were defined. Each period consisted of 10 days of adaptation to the diet and eight days of measurement. At the end of each experimental period, the cows changed treatments according to the assigned sequence: AB/BA (Kung-Jong 2016).

### Description of the production systems evaluated

The evaluated systems were:

#### SPI (Intensive Silvopastoral System = ISS + yacon)

Consisting of *C. clandestinus* grass, *T. diversifolia* shrubs for browsing in strips with furrows every 8 m, and scattered trees of (*Alnus acuminata* Kunth). The total area was 7984.82 m², and grazing was managed with daily strips covering an area of 221.80 m² (36 strips in total, with daily occupation/strip and 35 days of recovery). The cows in this group received balanced feed according to their nutritional requirements, with part of it replaced by *S. sonchifolius* silage.

#### MON (Monoculture system)

Monoculture of *C. clandestinus* with a total area of 7992 m². Pasture management was carried out with daily grazing strips covering an area of 222 m² (36 strips in total, with daily occupation/strip and 35 days of recovery). The cows in this group received only balanced feed according to their nutritional requirements.

### Animal selection

The animals were selected seeking the highest possible homogeneity between evaluations and within the groups/evaluation, using the following variables: live weight (kg), number of calvings, days in milk (DIM), average milk production during the last seven days before each evaluation (L/cow/day), and percentage of milk fat and protein (Table 1).

**Table 1:** Average characteristics of selected animals and sequence assignment in the two evaluations

Group	Weight (kg)	No. calving	Days in milk (DIM)	Milk production (L/cow/day)	Milk fat (%)	Milk protein (%)	Evaluation sequence
1	538.8 ± 60.15	2.05 ± 1.25	167.4 ± 18.5	24.6 ± 6.55	3.5 ± 0.55	3.05 ± 0.2	SPI (ISS + yacon) - MON
2	533.65 ± 49.8	2.15 ± 1.14	149.15 ± 45.05	22.05 ± 4.6	3.35 ± 0.45	3.05 ± 0.25	MON – SPI (ISS+ yacon)

### Nutritional management

For the evaluations carried out, the diets of the animals in the treatments were balanced, considering the requirements for crude protein, net lactation energy (NEL), Ca, and P (National Research Council 2001). The replacement of balanced feed with yacon silage in the SPI (ISS + yacon) was calculated at 20%. The chemical characterization of the feeds (Table 2) was performed using near-infrared spectroscopy (NIRS) at the Integrated Laboratory of Animal Nutrition, Biochemistry, and Pastures and Forages of the Faculty of Agricultural Sciences – University of Antioquia (Brand: FOSS; Model: DS 2500 F; Working software: ISIscan Nova DS2500F; Software version: 10.2.3.9) (Rivera Rivera et al. 2018; Pereira et al. 2023).

**Table 2:** Average nutritional content of the different components of the diet offered by treatment in the two evaluations

Treatment	Feed	DM	Ash	CP	Fat	CF	Sugars	NDF	ADF	Lig	Ca	P	GE
		%											Kcal/kg
SPI (ISS + yacon)	*C. clandestinus*	14.01	9.20	15.62	3.03	26.43	6.88	62.03	29.45	2.03	0.35	0.39	-
	Balanced feed	88.84	5.35	14.16	4.98	4.34	-	-	-	-	-	0.33	3834.50
	*T. diversifolia*	15.38	10.40	22.74	3.53	10.45	7.75	31.45	16.00	3.45	0.55	0.72	-
	Silage of *S. sonchifolius*	18.35	6.20	16.15	2.05	19.20	3.50	35.40	-	-	-	0.21	-
MON	*C. clandestinus*	14.68	10.28	14.75	3.03	24.13	7.10	59.55	27.13	1.75	0.32	0.39	-
	Balanced feed	88.84	5.35	14.16	4.98	4.34	-	-	-	-	-	0.33	3834.50

**DM**: Dry matter; **CP**: Crude protein; **CF**: Crude fiber; **NDF**: Neutral detergent fiber; **ADF**: Acid detergent fiber; **Lig**: Lignin; **Ca**: Calcium; **P**: Phosphorus; **GE**: Gross energy

The animals were grazed in accordance with the specifications described in the evaluated systems. Balanced feed was offered daily during the two milkings (5:00 a.m. and 2:00 p.m.). *S. sonchifolius* silage was prepared according to the protocol established by Lopera-Marín et al. (2024). It was offered in the paddock after each milking (3.5 kg/cow/milking) through individual plastic feeders to ensure 100% consumption in each experimental unit (Table 3).

**Table 3:** Average supply of balanced feed and silage of *S. sonchifolius* (fresh and dry matter) per treatment in the two evaluations

Treatment	Balanced feed		Silage of S. sonchifolius	
	kg/cow/day			
	FM	DM	FM	DM
SPI (ISS + yacon)	5.94	5.27	7	1.29
MON	7.18	6.38	0	0

**FM**: Fresh matter; **DM**: Dry matter

### Evaluated variables

#### Total dry matter intake (TDMI), production, and principal composition of milk

Grass and shrub intake was estimated in two intake tests, according to the methodology described by Lopera-Marín et al. (2025b), using the agronomic method of double sampling before and after grazing, considering a 30% remainder for grass (Haydock and Shaw 1975) and 5% for shrubs (Mejía-Díaz et al. 2017b). Consumption was estimated by the difference between the forage available before and after grazing. The consumption of balanced feed and yacon silage was estimated by supply and refusal.

Milk production was recorded individually and daily during each milking (5:00 a.m. and 2:00 p.m.). True-test Durespo meters manufactured by Tru-Test Group – Australia were used to quantify the volume (L/cow/day). The amount of milk (kg/cow/day) was calculated by multiplying the daily volume (L) by the apparent density of milk (1.032). Likewise, 4% fat-corrected milk (FCM) was calculated according to the standards established by the National Research Council (2001).

For the principal composition of milk, two samples were taken per animal per evaluation period. Each sample consisted of the milk produced by each cow in the two milkings (am – pm), mixing them according to the proportion of production obtained at each time. The samples collected were sent to the milk quality laboratory at the University of Antioquia (Medellín, Colombia) to determine the percentage of fat, protein, lactose, and total solids, as well as the milk urea nitrogen (MUN, mg/dL) using MilkoScan FT+ equipment (Fourier transform infrared; Foss, Hillerød, Denmark). The sanitary quality of the milk was determined by somatic cell count (SCC, x1000/mL) using flow cytometry (Fossomatic™ FC, Foss Electric, Hillerød, Denmark) from the samples collected.

#### Feed efficiency and energy efficiency

Energy efficiency (EE) was determined based on the ratio of NEL/L (net lactation energy required to produce one liter of milk) and NEL/FCM (net lactation energy required to produce one kilogram of fat-corrected milk). Feed efficiency (FE) was calculated from the ratio of FCM/kg TDM (kg of total dry matter consumed -TDM- to produce one kg of fat-corrected milk) (Angulo-Arizala et al. 2022a).

#### Economic analysis

For each system evaluated, the production cost per liter of milk was calculated, as well as the percentage distribution of the items that made up its structure, using the Quick Costs tool for Milk, Dual-Purpose, and Beef Production Systems (Gómez et al. 2018). The value of the monthly and annual savings from using strategic supplementation (*S. sonchifolius* silage) was calculated by projecting a scenario with 50 cows in production (Lopera-Marín et al. 2021; Angulo-Arizala et al. 2022a, b), considering the cost per kg of balanced feed, and silage (including transport) in both systems, and the difference when replacing balanced feed with silage.

### Statistical analysis

The information for the variables of Total dry matter intake (TDMI), production, and principal composition of milk was analyzed using ANOVA with the lme function of the nlme statistical package. The SCC was analyzed with ANOVA using the glmer function of the lme4 statistical package. All information was analyzed using R-Project software version 4.4.1 (R Core Team 2024). For all variables evaluated, treatments (SPI and MON) were considered fixed effects, and evaluations (1 and 2), periods per evaluation (periods I and II), and cow groups (G1 and G2) were considered random effects. Covariates (live weight -kg-, number of calvings, days in milk, and last milk production -L/cow/day-) were included in the analysis to consider adjustments to the models used in the ANOVA. In case of rejection of the null hypothesis, Tukey’s test (*p < 0.05*) was used to separate the means.

## Results

### Total dry matter intake (TDMI) and principal composition of milk

There were differences in TDMI (*p < 0.05*) in favor of the SPI (ISS + yacon); however, production (Table 4) and principal composition of milk (Table 5) were similar between the two treatments (*p > 0.05*). The SCC showed a significant difference (*p < 0.05*) in favor of the SPI (Table 5).

**Table 4:** Dry matter intake and milk production in the systems evaluated

Variable	Treatment		RMSE	*p*-value
	SPI (ISS + yacon)	MON		
TDMI, kg/cow/day	22.78 ± 0.35ª	18.24 ± 0.35^b^	13.294	0.0028
Production, L/cow/day	20.70 ± 0.54	21.39 ± 0.54	2.1754	0.2030
FCM, kg/day	21.28 ± 0.64	21.96 ± 0.64	1.2196	0.5248

**TDMI**: Total dry matter intake; **FCM**: Fat-corrected milk; **RMSE**: Root Mean Square Error

Different letters in the same row indicate a significant difference (*p < 0.05*)

**Table 5:** Principal composition of milk in the systems evaluated

Variable	Treatment		RMSE	*p*-value
	SPI (ISS + yacon)	MON		
Fat, %	3.83 ± 0.28	3.79 ± 0.28	0.7202	0.8603
Fat, kg/día	0.78 ± 0.04	0.80 ± 0.04	3.6764E-07	0.6410
Protein, %	3.06 ± 0.05	3.21 ± 0.05	0.1905	0.1375
Protein, kg/día	0.63 ± 0.03	0.67 ± 0.03	0.0557	0.1091
Lactose, %	4.50 ± 0.05	4.49 ± 0.05	0.07951	0.3508
Lactose, kg/día	0.94 ± 0.05	0.94 ± 0.05	0.1342	0.5893
TS, %	12.04 ± 0.30	12.20 ± 0.30	0.9512	0.6805
TS, kg/día	2.49 ± 0.08	2.57 ± 0.08	0.2436	0.2856
MUN, mg/dL	14.10 ± 0.66	15.17 ± 0.66	1.2137	0.2036
SCC, x1000/mL	157 ± 64.2^b^	212 ± 86.5^a^	162.2195	< 0.0001

**TS**: Total solids; **MUN**: Milk urea nitrogen; **SCC**: Somatic cell count; **RMSE**: Root Mean Square Error

Different letters in the same row indicate a significant difference (*p < 0.05*)

### Feed efficiency and energy efficiency

Table 6 shows the results for energy efficiency and feed efficiency. Although the silvopastoral system (SPI) showed lower feed efficiency (0.90 vs. 1.16) and lower energy efficiency (a higher NEL/milk produced ratio, 1.37 vs. 0.98), this was not reflected in a significant difference in milk production or milk compositional quality.

**Table 6:** Energy and feed efficiency of the systems evaluated

Variable	Treatment		RMSE	*p*-value
	SPI (ISS + yacon)	MON		
Energy Efficiency (EE), NEL/L	1.37 ± 0.06^a^	0.98 ± 0.06^b^	0.16791	0.0078
Energy Efficiency (EE), NEL/FCM	1.37 ± 0.04^a^	0.98 ± 0.04^b^	0.1583445	0.0067
Feed Efficiency (FE), FCM/kg TDMI	0.90 ± 0.03^b^	1.16 ± 0.03^a^	0.1194607	0.0098

**EE (NEL/L)**: Energy Efficiency (Net Lactation Energy required to produce 1 L of milk); **EE (NEL/FCM)**: Energy Efficiency (Net Lactation Energy required to produce 1 kg FCM); **FE (FCM/kg TDMI)**: kg of TDMI to produce 1 kg FCM

**RMSE**: Root Mean Square Error

Different letters in the same row indicate a significant difference (*p < 0.05*)

### Economic analysis

The scenario was favorable for the SPI, showing a reduction in production costs of 0.013 USD per L of milk compared to the MON. In the percentage distribution of costs in the systems evaluated, the difference was mainly due to the balanced feed. In the MON system, this item represented 47.8%, and by replacing 1.29 kg of DM with *S. sonchifolius* silage in the SPI, this item was reduced by 6.2%, resulting in a net profit margin of 26.67% in the SPI and 22.67% in the MON. When projecting feed costs for 50 dairy cows, a monthly saving of USD 411.43 was found in the SPI, and an annual saving of USD 4937.13 (Table 7).

**Table 7:** Economic analysis of the systems evaluated

System	Item	Total (USD $)
SPI (ISS + yacon)	Production costs/L of milk	0.236
	kg of balanced feed	1.81
	kg of yacon silage (including transportation)	0.45
	Cost of balanced feed + yacon silage/cow/day	2.26
	Difference between balanced feed + yacon silage/cow/day	0.27
	Cost of balanced feed + yacon silage/cow/month	67.79
	Cost of balanced feed + yacon silage/50 cows/month	3389.40
	**Savings with strategic supplementation/month**	**411.43**
	**Savings with strategic supplementation/year**	**4937.13**
MON	Production costs/L of milk	0.248
	kg of balanced feed	2.53
	kg of yacon silage (including transportation)	0.00
	Cost of balanced feed + yacon silage/cow/day	2.53
	Difference between balanced feed + yacon silage/cow/day	0.00
	Cost of balanced feed + yacon silage/cow/month	76.02
	Cost of balanced feed + yacon silage/50 cows/month	3800.82
	**Savings with strategic supplementation/month**	**0.00**
	**Savings with strategic supplementation/year**	**0.00**

## Discussion

### Total dry matter intake (TDMI) and principal composition of milk

The similarity in both production and principal composition of milk in the SPI can be explained by the rumen N provided by *T. diversifolia* and the energy content in the diet from *S. sonchifolius* silage and *C. clandestinus* grass, generating microbial protein that passes to the small intestine, where it is used in biochemical processes that maintain or increase milk volume and composition (López et al. 2015). This indicates that the balance of the diets in both treatments was correct, and the replacement of balanced feed with yacon silage in the SPI successfully fulfilled the nutritional requirements of the cows. Studies in which 25% of the diet is replaced with *T. diversifolia* forage show results without statistical differences (in terms of production and principal composition of milk), which are consistent with those reported in this research (Arias-Gamboa et al. 2018; Gallego Castro et al. 2017).

The values found for the SCC are within the minimum threshold established by Colombian standards for raw milk (Instituto Colombiano de Normas Técnicas y Certificación 2018). Milk from the SPI is classified as having excellent sanitary quality due to the absence of inflammation in the mammary glands (clinical and subclinical mastitis) and the reduction in costs resulting from the non-use of veterinary drugs to control these conditions (Alvarado et al. 2019; Jurado-Gámez et al. 2021; Stocco et al. 2020). An evaluation of these systems reported a lower SCC (*p < 0.05*) (67.26 × 1000/mL) compared to *C. clandestinus* monoculture (125.35 × 1000/mL) (Cardona-Iglesias et al. 2019). When *T. diversifolia* meal was included in 25% of the diet of dairy cows, the SCC was lower (*p < 0.05*) (44.08 × 1000/mL) compared to the control treatment (177.08 × 1000/mL) (Gallego Castro et al. 2017).

The reduction in SCC may be related to the overall state of the mammary gland’s immune system, which can be positively modulated in more diverse and sustainable systems. It is well known that SCC increases in response to intramammary infections, but it can also reflect the cow’s systemic inflammatory state. The diet in silvopastoral systems is usually richer in effective fiber, natural antioxidants, and bioactive compounds, which can modulate the inflammatory response and improve innate immune function (Knegsel et al. 2014; Wall et al. 2018).

In this regard, consumption of *T. diversifolia* provides secondary metabolites with anti-inflammatory and antimicrobial properties, such as tannins, sesquiterpene lactones, flavonoids, and saponins (Reena et al. 2012; Valdivia Avila et al. 2018; Rivera et al. 2018; Cardona-Iglesias et al. 2017), which could contribute to reducing bacterial load or subclinical inflammation in the udder. These compounds have demonstrated inhibitory activity against relevant pathogens, such as *Staphylococcus aureus* and *Proteus vulgaris*, even under in vitro conditions (Gutierrez et al. 2013; Mabou Tagne et al. 2018; Londoño et al. 2020). Likewise, significant inhibition of carrageenan-induced edema has been reported, supporting its anti-inflammatory potential (Sijuade et al. 2016).

Complementarily, yacon (*Smallanthus sonchifolius*), another Asteraceae, offered to cows in this study in the form of silage, contains secondary metabolites (such as phenols and chlorogenic acids) and fructooligosaccharides (FOS) that are recognized for their prebiotic effects. These compounds promote the growth of beneficial bacteria, such as *Lactobacillus* and *Bifidobacterium*, increase the production of short-chain fatty acids, enhance intestinal mucosal health, and improve mineral absorption (Lima et al. 2018; Romualdo et al. 2023). In animal models, FOS extracted from yacon has shown anti-inflammatory and immunomodulatory activity (Choque Delgado et al. 2012). However, it is essential to note that this study did not directly determine the content of inulin or secondary metabolites in Mexican sunflower or yacon; therefore, these effects should be considered preliminary hypotheses and verified in future studies.

It should also be considered that the quality of the diet at the ruminal level significantly influences the microbiota and the immune system, with potential implications for udder health. More natural diets, with lower concentrate inclusion, have been shown to promote a more balanced ruminal microbiota, characterized by a greater abundance of beneficial bacteria and a lower presence of potential pathogenic microorganisms in milk (Wang et al. 2022). In contrast, diets high in concentrate, common in intensive systems, have been associated with an imbalance in the rumen microbiota, activation of inflammatory pathways such as NF-κB, and immunosuppression, conditions that could favor an environment more susceptible to mammary alterations, such as elevated SCC or subclinical inflammatory processes (Wang et al. 2024). In addition, a direct relationship between gastrointestinal microbiota imbalance and mammary inflammation has been demonstrated, reinforcing the gut-mammary gland axis hypothesis (Jie et al. 2025).

Additional studies with other secondary plant metabolites support this line of thinking. Walkenhorst et al. (2019) observed a 15% reduction in SCC using an additive composed of medicinal herbs, while Prapaiwong et al. (2023) reported a significant decrease in cows supplemented with hydrolyzable tannins from *Castanea sativa*. These findings suggest that bioactive compounds, integrated into more balanced and diverse dietary systems, may exert synergistic effects on udder health.

Finally, it cannot be ruled out that characteristics specific to the SPI, such as forage diversity or a more gradual release of energy, may have contributed to preventing metabolic excesses and promoting a more balanced and less pro-inflammatory state in cows. Although production and principal composition of milk did not differ between treatments, the improved udder health reflected in lower SCC could represent a key advantage in terms of animal welfare and milk quality, especially under the conditions in the Colombian tropical highlands.

### Feed efficiency and energy efficiency

The variations in efficiency could be explained by the higher total dry matter intake in the SPI, particularly dry matter from forage, resulting from the partial replacement of concentrate. This higher forage intake allowed net energy and protein intakes to be maintained, compensating for the lower energy density of the forage components (*T. diversifolia* or *S. sonchifolius* silage). This adjustment may be related to the good palatability and digestibility reported for these species, which favor ruminal activity and stimulate voluntary intake (Mejía Díaz et al. 2017a, 2017b; Lopera-Marín et al. 2024). Therefore, although the SPI showed apparently lower conversion efficiency, it managed to maintain productive performance with less dependence on concentrates, which represents an advantage in terms of sustainability and the use of local feed resources (Cantalapiedra-Híjar and Molina-Alcaide 2021).

### Economic analysis

Evaluations of these systems found production costs per liter of milk to be USD 0.24 and USD 0.26, with net income per hectare per year increasing by up to 50% (Mejía-Díaz et al. 2017b). Studies conducted in conventional and silvopastoral systems in dairy regions of the high tropics in Colombia showed production costs per liter of milk of USD 0.236 and USD 0.185, respectively, with net profit margins of 26.5% and up to 55.6%, respectively (Lopera Marín et al. 2021). In trials using *T. diversifolia* silage and supplementing for 50 dairy cows, a monthly savings of USD 281 was found in feeding these cows (Angulo-Arizala et al. 2022a, b).

Evaluations in different silvopastoral systems show improvements in net income (close to USD 500) compared to monoculture systems (Sandoval et al. 2022). In an evaluation of partial replacement of concentrated feed with *Sambucus peruviana* shrub foliage for a herd of 50 dairy cows, this resulted in an additional monthly income of USD 133 (Durana et al. 2022). The methodology used in several of the studies cited above, when modeling cost reduction and net income improvement for 50 cows, is quite reliable under Colombian conditions, since the average number of cows per producer is between 10 and 16, and only 5% of milk producers have more than 50 cows in production (IFCN 2014).

The reduction in costs without affecting the quantity and quality of production found in this research, thanks to the combination of local natural feeds that reduced concentrated use by 20%, would allow dairy farmers in the high tropics to be in the most competitive segment, which has high productivity and low costs (< USD 0.26), represented by only 2.5% of farms, which in turn generate 15% of the milk volume in Colombia (Carulla and Ortega 2016). A recent evaluation showed that an SSP offers superior profitability and more efficient financial performance in the medium term, as well as a favorable benefit-cost (B/C) ratio of 2.05, compared to 1.115 in the control system (Narváez-Herrera et al. 2025).

In Costa Rica, the use of *T. diversifolia* as a supplement for Jersey dairy cows resulted in savings of USD 0.25 and USD 0.52 per animal per day in treatments where 25% and 50% of the concentrated feed was replaced, respectively. Replacing 25% of the balanced feed with *T. diversifolia* forage did not affect the production or nutritional quality of milk in Jersey cows, resulting in savings of 9.06% due to lower costs (Arias-Gamboa et al. 2018). Some economic models in silvopastoral systems show better internal rates of return (IRR) and higher profitability. These improve when arrangements include strips of timber trees, where IRRs between 12.18% and 28.46% can be obtained, depending on the level of investment based on the number of trees established (Ospino et al. 2022).

It is concluded that the silvopastoral system (SPI) is a viable alternative for dairy production systems, as it maintains milk yield and principal milk composition similar to a monoculture system, while reducing concentrate use. The SPI also improves milk hygienic quality with a lower somatic cell count. The inclusion of locally available forages, such as Mexican sunflower and yacon, ensured adequate nutrient intake. Economic analysis favored the SPI, since projecting the conditions of the SPI with 50 dairy cows results in significant monthly and annual savings in feed alone, representing a relevant economic benefit for small-scale producers, who make up the majority of dairy farms in Colombia. Therefore, silvopastoral systems in the high tropics, when strategically supplemented, are a viable strategy for maintaining milk production and reducing production costs.

## Data Availability

This is not applicable.
